# Dnajb8, a Member of the Heat Shock Protein 40 Family Has a Role in the Tumor Initiation and Resistance to Docetaxel but Is Dispensable for Stress Response

**DOI:** 10.1371/journal.pone.0146501

**Published:** 2016-01-11

**Authors:** Masamichi Yamashita, Yoshihiko Hirohashi, Toshihiko Torigoe, Hiroki Kusumoto, Aiko Murai, Tomohiro Imagawa, Noriyuki Sato

**Affiliations:** 1 Department of Pathology, Sapporo Medical University School of Medicine, South-1 West-17, Chuo-ku, Sapporo 060–8556, Japan; 2 Department of Veterinary Diagnostic Imaging, School of Veterinary Medicine, Faculty of Agriculture, Tottori University, Tottori 680–8553, Japan; Cedars-Sinai Medical Center, UNITED STATES

## Abstract

Cancer stem-like cells (CSCs)/cancer-initiating cells (CICs) are defined by their abilities of tumor initiation, self-renewal and differentiation. In a previous study, we showed by gene knockdown using siRNA and gene overexpression experiments that Dnaj (Hsp40) homolog, subfamily B, member 8 (DNAJB8), a role in the maintenance, of renal cell carcinoma CSCs/CICs. In the present study, we established Dnajb8 knockout (KO) renal cell carcinoma (RCC) line cells (RenCa cells) and analyzed the cells to confirm the function of Dnajb8 in RCC CSCs/CICs. Dnajb8 KO cells showed reduced ratios of side population cells and reduced sphere forming ability. An in vivo single cell tumor initiation assay revealed that the numbers of CSCs/CICs were 3 in 4 wild-type RenCa cells and 1 in 4 Dnajb8 KO cells. Dnajb8 KO cells showed sensitivity to Docetaxel. On the other hand, Dnajb8 KO cells did not show any sensitivities to stresses including low pH, low glucose, heat shock and sensitivity to cisplatin. The results indicate that Dnajb8 has a role in tumor initiation, side population ratio and sphere formation but it is dispensable for stress responses.

## Introduction

Cancer stem-like cells/cancer-initiating cells (CSCs/CICs) are defined by their ability of tumor initiation, self-renewal and differentiation [1, 2]. CSCs/CICs are resistant to stresses including stresses from chemotherapy and radiotherapy [3]. It is thus thought that CSCs/CICs are responsible for relapse after treatment and distant metastasis, and eradication of CSCs/CICs is essential to cure cancer. CSCs/CICs can be isolated and analyzed by several methods [4–6]; however, the molecular aspects of CSCs/CICs are still elusive.

Dnaj (Hsp40) homolog, subfamily B, member 8 (DNAJB8) belongs to the heat shock protein (HSP) 40 family and has a role in suppression of misfolded toxic protein aggregation [7, 8]. Recently, it has been reported that some members of the HSP40 family are related to the development and metastasis of cancers and that their expression was detected in breast cancer stem cells [9]. We reported that DNAJB8 is expressed preferentially in CSCs/CICs derived from renal cell carcinoma (RCC) and colorectal cancer and that DNAJB8 has an important role in the maintenance of CSCs/CICs [10, 11]. However, the mechanism by which DNAJB8 affects the maintenance of cancer stem cells has not been clarified.

Functions of genes have been analyzed by gene targeting including gene knockout and gene knockdown. Gene knockdown by siRNAs is easy and the cost is comparatively low, and we have also analyzed the function of DNAJB8 by gene knockdown using siRNAs [10]. However, gene expression does not completely disappear and functional analysis of the gene cannot proceed stably. Recently, the CRISPR/Cas9 system was developed for convenient genome editing[12]. Using this system, an optional target genome sequence can be cut and continuously induced for gene knockout (KO) caused by a frameshift or knock-in of the optional sequence. In this study, we established novel DNAJB8 KO cells by using the CRISPR/Cas9 system, and we analyzed the properties of the KO cells.

## Materials and Methods

### Ethics statement

Mice were maintained and experimented on in accordance with the guidelines of and after approval by the Committee of Sapporo Medical University School of Medicine, Animal Experimentation Center under permit number 08–006. We monitored the physical conditions of the mouse every other day, and one mice was found dead in unknown reason by autopsy. All studies were approved by the Institutional Review Board (IRB) of Sapporo Medical University Hospital.

### Cell line

The murine RCC cell line RenCa of BALB/c mouse origin was maintained in RPMI1640 (Sigma-Aldrich, St Louis, MO, USA) supplemented with 10% FBS. Dnajb8-overexpressed RenCa cells were established previously [10].

### CRISPR/Cas9 system

Knockout of the Dnajb8 gene was carried out by using a GeneArt^Ⓡ^ CRISPR Nuclease Vector Kit (Life Technologies, Carlsbad, CA, USA). A target sequence was inserted into the CRISPR nuclease vector, and the vector was transduced into One Shot^Ⓡ^ Top 10 (Life Technologies). The plasmid including the CRISPR nuclease construct was refined using a Qiafilter Plasmid Maxi Kit (Qiagen, Valencia, CA, USA), and RenCa cells were transduced with the vector using lipofectamine 2000 (Life Technologies) following the manufacturer’s protocol. The transduced cells were sorted in single cells using a BD FACS Aria II Cell-Sorting System (BD, Franklin Lakes, NJ, USA).

### Polymerase chain reaction (PCR), sequence analysis and RT-PCR

To confirm gene knockout of Dnajb8, we analyzed genome DNA by PCR and DNA sequencing. The genome DNA was extracted using a QIAamp DNA Mini Kit (Qiagen) according to the manufacturer’s protocol. PCR was performed in 20 μL of PCR mixture containing 1 μL of genome DNA, 0.1 μL of Taq polymerase (Qiagen) and 4 pmol of primers. The PCR mixture was initially incubated at 94°C for 2 min followed by 35 cycles of denaturation at 94°C for 15 sec, annealing at 58°C for 30 sec and extension at 7°C for 30 sec. The primer pairs used for PCR analysis were 5’-ACGGGGAGGATGGAAAGCTA-3’ and 5’-AGACTTCGTAGGCTTCGGAAA-3’ with an expected PCR product size of 305 bp. Genome sequence analysis was performed in 10 μL of PCR mixture containing 1 μL of PCR product extracted, 2 μL of Big dye and 4 pmol of forward primer using a BigDye^Ⓡ^ Terminator v1.1 Cycle Sequencing Kit (Life Technologies). The PCR product was extracted using ethanol precipitation, and DNA sequencing was performed using a 3130 Genetic Analyzer (Applied Biosystems, Foster City, CA, USA).

Total RNAs (tRNA) were isolated from RenCa, Dnajb8 overexpressed RenCa (RenCa/Dnajb8)[10], RenCa Dnajb8 KO #1–2 and RenCa Dnajb8 KO #4–1 cells using an RNeasy Mini Kit (Qiagen, Valencia, CA). Complementary DNA (cDNA) was synthesized and RT-PCR was performed as described previsouly [13]. Primer pairs used for RT-PCR analysis were 5’-GATCAGCATGTACCTCCCCG-3’ and 5’-CGCCCTCAGGTTTTCTCTGT-3’ for *Sox2*, 5’-CTGGGCTTAAAGTCAGGGCA-3’ and 5’-AGGTTTGCTCTGGCACCAAT-3’ for *Nanog*, 5’-TCACATGAAGCGACTTCCC-3’ and 5’- CCCGGATCGGATAGCTGAAG-3’ for *Klf4*, 5’-TCACCAGAGGGATGGACTGA-3’ and 5’-ATTGGTGGTTAGCACTGGGG-3’ for *Bmi1*, and 5’-GGGAAGCCCATCACCATCTT-3’ and 5’-GTGTAGCCCAAGATGCCCTT-3’ for *Gapdh*. *Gapdh* was used as an internal positive control.

### Western blot analysis

The cell lysate with SDS sample buffer was separated by denaturing SDS-PAGE. Separated proteins were transferred onto nitrocellulose membranes and probed with an anti-DNAJB8 antibody that we established previously[10]. β-Actin was used a loading control and was detected with a mouse mAb (Sigma-Aldrich). Anti-DNAJB8 antibody was used at 250-times dilution, and anti-β-actin antibody was used at 2,000-times dilution.

### Cell growth analysis and chemo resistance

To compare the cell growth rates, 10^4^ cells were plated in a 6-well plate and cultured in RPMI1640 (Sigma-Aldrich) supplemented with 10% FBS in a 5% CO_2_ incubator for 1, 3 and 5 days, and the number of cells was counted by Countess® (Life Technologies).

To address sensitivities for chemotherapeutic reagents, RenCa, Danjb8 overexpressed RenCa (RenCa/Dnajb8), Dnajb8 KO cells (#1–2 and #4–1) were incubated with Docetaxel (Wako chemicals, Osaka, JAPAN) or Cisplatin (Wako chemicals) at several concentrations for 96 hrs. The viability of cells were addressed using WST-8 reagent (Dojindo Molecular Technologies, Tokyo, JAPAN).

### Sphere forming assay

A sphere forming assay was performed as described previously [14]. One thousand cells were plated in Ultra Low Attachment 6-well plates (Corning Incorporated Life Sciences, Acton, MA, USA) and cultured in Dulbecco’s modified Eagle’s medium//F12 (Life Technologies) supplemented with 10 ng/mL epidermal growth factor, 15 ng/mL basic fibroblast growth factor and N2 Supplement with Transderrin (Wako Pure Chemical Industries, Ltd., Osaka, Japan) at 100-times dilution in a 5% CO_2_ incubator for 22 days, and the numbers of spheres were counted under a microscope in all fields and then the average was calculated.

### Side population analysis

SP analysis was performed as described previously [10, 11]. The cells were labeled with Hoechst 33342 dye (Life Technologies) for 90 min at a concentration of 3 μg/mL with or without Verapamil (Sigma-Aldrich), which is an inhibitor of ATP-binding cassette (ABC) transporters, at a concentration of 75 μM. Cell were counterstained with 1 μg/mL propidium (Sigma-Aldrich) for labeling dead cells. Viable cells were sorted using a FACS.

### Stress resistance analysis

To assess the stress resistance of Dnajb8 KO cells, KO cells were cultured in an environment of low pH, low level of nutrition and high temperature. For low pH (pH 6.0), hydrochloric acid was added to RPMI1640 medium supplemented with 10% FBS. For a low level of nutrition, no glucose RPMI1640 medium (Wako, Osaka, Japan) supplemented with 10% FBS was used. Ten thousand cells were plated in a 6-well plate using RPMI1640 medium and the medium was changed to each adjustment medium for 24 hr. After incubation, the medium was changed to normal PRMI1640 medium and cells were counted after 5 days. For a high temperature, cells were incubated at 45°C for 30 min, 1 hr and 2 hr at 24 hours after cells had been plated, and cells were counted after 5 days.

### Poly-Q aggregation formation assay

Cells were transfected with ataxin-3 cDNA with extended glutamine residues (Ataxin-3 T-Q82). The cells were cultured on glass cover slips (Fisher Scientific, Pittsburgh, PA, USA) for more than 24 hr and fixed in 1% paraformaldehyde for 30 min. The cover slips were washed 3 times with PBS, mounted using VECTORSHIELD mounting medium with DAPI (VECTOR LABORATORIES, INC., Burlingame, CA), and visualized by confocal laser scanning microscopy (LSM510, Carl Zeiss, Tokyo, Japan).

### Transplantation in mice

All procedures using mice were carried out in accordance with institutional protocol guidelines at Sapporo Medical University School of Medicine. BALB/c female mice were purchased from Clea Japan (Tokyo, JAPAN) at the age of 6 to 8 weeks. RenCa cells and Dnajb8 KO RenCa #1–2 cells were mixed with matrigel (BD) at a 1:1 volume and injected subcutaneously into the flank of mice. Tumor size was assessed weekly using a caliper and calculated using the following formula: tumor size (mm^3^) = (longest diameter × shortest diameter^2^)/2. For single cell injection, wild RenCa and Dnajb8 KO RenCa cells were single-cell sorted by FACS Aria II into 96-well plates. Single cells in the 96-well plates were mixed with matrigel at a 1:1 volume and injected into the backs of mice. The total number of injection points was 20 in each group, and tumor initiation was observed. The viability of single-sorted cells was confirmed by further culture in 96-well plates. All mouse were sacrificed by CO_2_ under anesthesia after experiments.

## Results

### Gene knockout of *Dnajb8* by the CRISPR/Cas9 system

**Fig 1:**
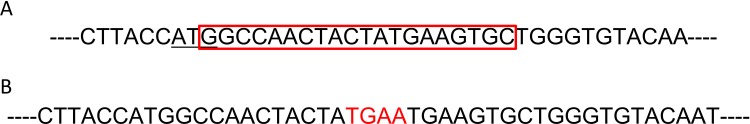
Design of the target sequence for CRISPR/cas9. (A) Target sequence of CRISPR/Cas9 construct. The bar indicates the start codon of Dnajb8. (B) The design of ssDNA. Stop codon (TGAA) is inserted.

Genome editing using the CRISPR/Cas9 system is a powerful tool for gene engineering [15]. In a previous study, we found that DNAJB8 is expressed in CSCs/CICs derived from mouse and human renal cell carcinoma (RCC) line cells RenCa and ACHN, and we found by using siRNA technology that DNAJB8 has a role in the maintenance of RCC CSCs/CICs [10]. However, gene knockdown using siRNAs is temporary and incomplete. We thus performed gene knockout of Dnajb8 using the CRISPR/Cas9 system to address the function of Dnajb8 in cancer cells. In this study, we used two approaches to knockout Dnajb8; (A) gene knockout by a frame shift and (B) knock-in of the stop codon in the coding region of Danjb8. For knockout of a frameshift, we designed a 20-base-pair sequence just downstream of the start codon (Fig 1A). For stop codon knock-in, we designed a 124-base-pair single-strand DNA (ssDNA) that contains 4 base pairs including a stop codon, are we inserted it in the middle of the target sequence of CRISPR/Cas9 (Fig 1B).

**Fig 2:**
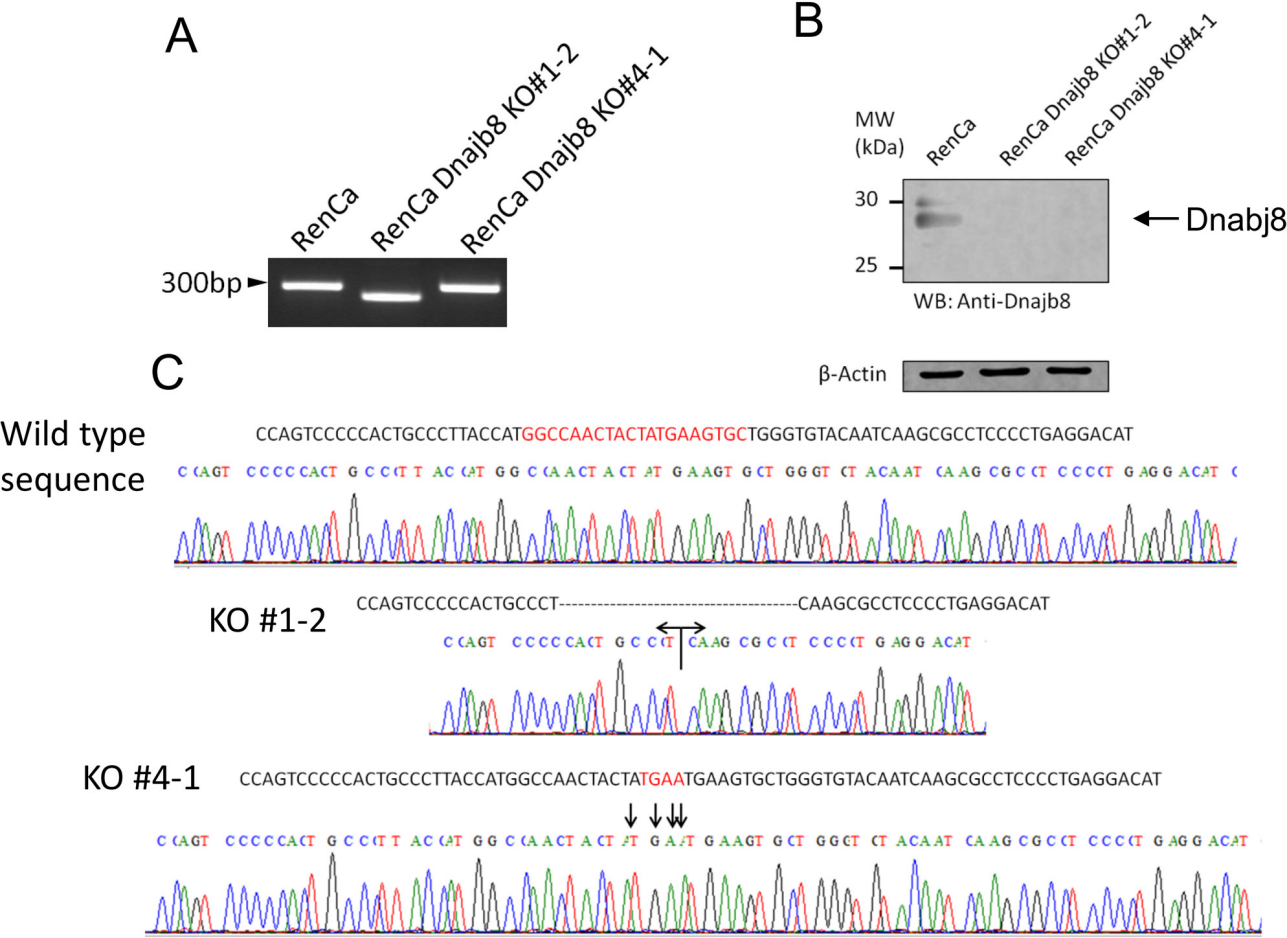
Establishment of Dnajb8 KO cells. (A) Amplification of the Dnajb8 region targeted by CRISPR/Cas9. (B) Western blot analysis of DNAJB8 protein. (C) DNA sequence of Dnajb8 KO cells.

The CRISPR/Cas9 construct alone or with ssDNA was transfected into RenCa cells, and then the transfection-positive cells were sorted into 96-well plates and cloned by flow cytometer. We obtained 31 independent clones, and we performed PCR analysis and DNA sequence analysis to screen for Dnajb8 knockout clones. One clone, clone #1–2, showed an apparent band shift of the Dnajb8 gene (Fig 2A), and sequence analysis of clone #1–2 revealed a 38-base-pair deletion, indicating a frame shift of DNAJB8 protein (Fig 2C). Sequence analysis of another clone, clone #4–1, revealed a stop codon insertion just downstream of the start codon as designed in ssDNA (Fig 2C). Protein deletion of DNAJB8 was confirmed by Western blot analysis in clone #1–2 and clone #4–1 cells (Fig 2B). Thus, both clone #1–2 and clone #4–1 cells were shown to be Dnajb8 knockout RenCa cells, and they were used for further analysis in the following experiments.

### *In vitro* evaluation of Dnajb8 KO cells

**Fig 3:**
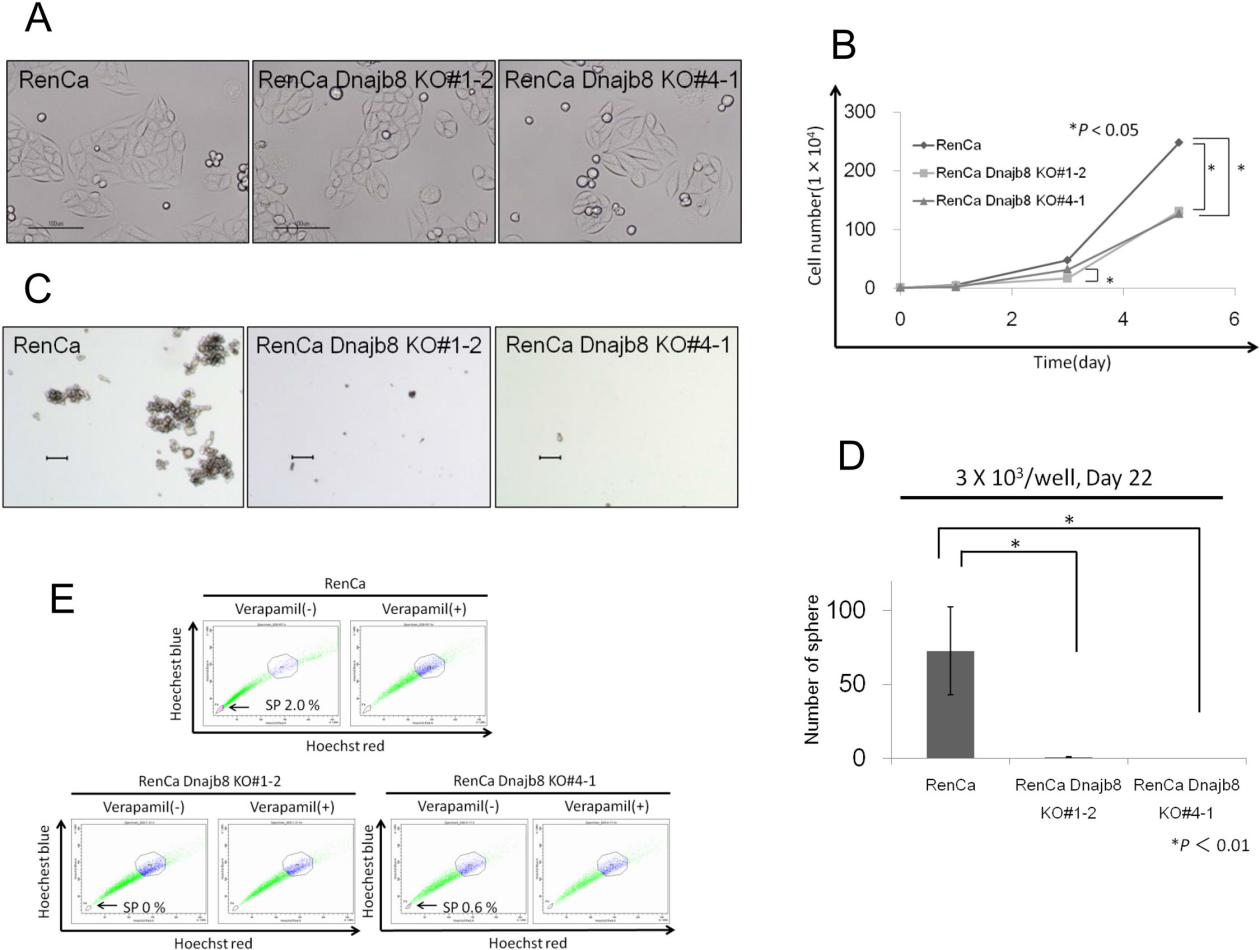
In vitro analysis of Dnajb8 KO cells. (A) Representative images of WT cells and Dnajb8 KO cells. Bars indicate 100 μm. (B) Cell proliferation assay of WT cells and Dnajb8 KO cells. 10^4^ cells were cultured and the number of cells was counted at 1, 3 and 5 days. Data represent means ± SD. The difference between WT cells and two Dnajb8 KO cells was examined for statistical significance using Student’s *t*-test. (C) Representative images of spheres derived from WT cells and Dnajb8 KO cells. Bars indicate 100 μm. (D) Sphere formation assay. To assay sphere formation efficiency, 10^3^ cells were cultured in floating condition for 22 days and the numbers of spheres were counted under a microscope in all fields. (E) Side population (SP) assay. WT cells and Dnajb8 KO cells were stained with Hoechst 33342 dye with or without verapamil and analyzed using a FACSAria II cell sorter.

Phenotypes of Dnajb8 KO cells were evaluated in comparison with wild-type (WT) cells as a control. The shape of Dnajb8 KO cells was an epithelial-like form, similar to that of WT cells (Fig 3A). The growth rates of Dnajb8 KO cells and WT cells were compared, and Dnajb8 KO cells showed significantly slower growth than that of WT cells (Fig 3B).

Since CSCs/CICs have the ability to form spheres in a floating culture condition [16], a sphere forming assay was performed. WT cells showed sphere formation, but Dnajb8 KO cells showed no sphere formation (Fig 3C and 3D). The side population (SP) assay is another method for examining CSCs/CICs [17], and we performed SP assays using WT cells and Dnajb8 KO cells (Fig 3E). To evaluate the effect of Dnajb8 on SP cells, WT cells and Dnajb8 KO cells were analyzed by SP assays. The ratio of SP cells in WT cells was 2.0%, whereas the ratio of clone #1–2 cells was 0% and that of clone #4–1 cells was 0.6% (Fig 3E). Stem cell-related gene products including Sox2 and Bmi-1 have roles in the maintenance of CSCs/CICs [18, 19], we thus analyzed the expressions of stem cell related genes in Dnajb8 KO cells (S1 Fig). The expressions of Sox2 and Nanog were not detected in RenCa WT cells, Dnajb8-overexpressed RenCa cells (RenCa/Dnajb8) and Dnajb8 KO cell (#1–2 and #4–1). And there are no differences in the expression levels of Klf4 and Bmi-1 indicating that these stem cell-related genes are not involved in the function of Dnajb8.

### Dnajb8 is dispensable for stress responses

**Fig 4:**
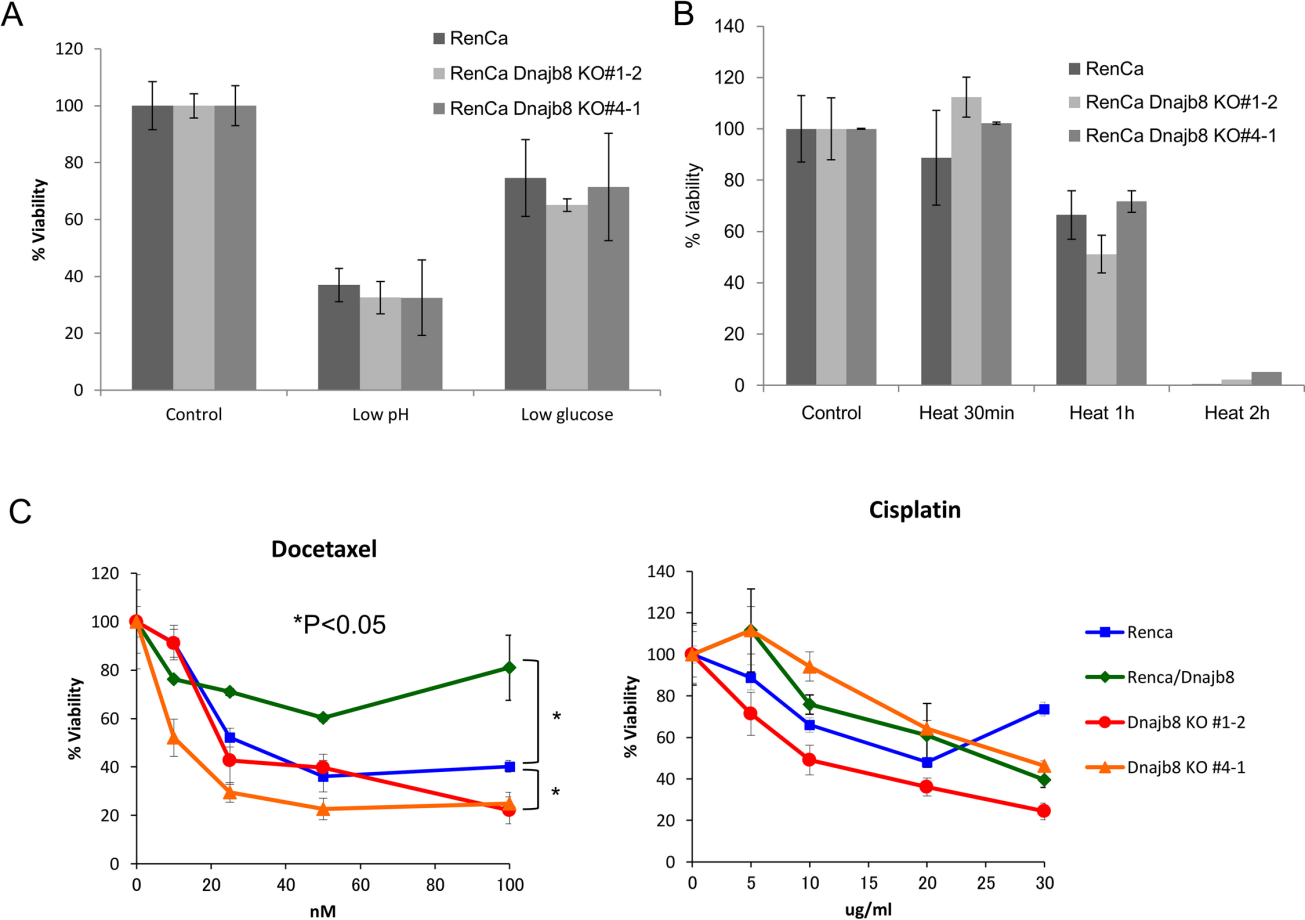
Analysis of stress response. (A) Low pH stress and low glucose stress. Ten thousand cells were cultured for 5 days and the number of cells was counted. Data represent the percent of alive cell rates compared with control groups. (B) Responses to heat shock stresses. Ten thousand cells were cultured for 5 days after heat shock stress, and the number of cells was counted. Data represent the percent of alive cell rates compared with control groups. (C) Chemo-resistance. Eight thousand cells were cultured for 4 days in chemotherapeutic reagents (Docetaxel or Cisplatin). The percentage of viability were addressed using WST-8 reagents. Data are shown as means ± SD. Differences between groups were examined for statistical significance by Student's t-test.

Dnajb8 belongs to HSP40 family, and we thus addressed the functions of Dnajb8 in stress responses including heat shock, low pH and low glucose[20]. The alive cell rates under low pH and low glucose were examined by cell counts, and there were no significant difference between WT cells and KO cells (Fig 4A). The alive cell rates were decreased according to the length of heat stress (Fig 4B). However, there was no significant difference between WT cells and KO cells. CSCs/CICs are resistant to chemotherapeutic reagents by several molecular mechanisms [3]. We thus addressed the chemo-sensitivities using RenCa, Dnajb8 KO cells and Dnajb8-overexpressed cells (RenCa/Dnajb8). Interestingly, RenCa/Dnajb8 cells showed resistance to docetaxel, but did not to cisplatin. Dnajb8 KO cells showed higher sensitivity to docetaxel, but did not to cisplatin (Fig 4C). These results indicate that Dnajb8 has a role in resistance to docetaxel, but not to cisplatin.

In the previous study, Dnajb8 was described to has a role in suppression of toxic polyglutamine (polyQ) protein aggregation [7]. We identified other DNAJ protein, DNAJC8 has a role in suppression of toxic polyQ protein aggregation (unpublished data). We therefore addressed the suppression of protein aggregation using Dnajb8 KO cells. Ataxin-3 gene carrying 82 polyglutamine residue fused with GFP protein are constructed previously (Ataxin-3 T-Q82) and we confirmed that overexpression of Ataxin-3 T-Q82 gene make protein aggregation in SH-SY5Y human neuroblastoma cells (unpublished data). We transfected Ataxin-3 T-Q82 gene into RenCa WT cells, RenCa/Dnajb8 KO cells (#1–2 and #4–1) and Dnajb8 overexpressed cells (RenCa/Dnajb8). However, no cells made protein aggregation in all cell lines, and we could not address the suppression of protein aggregation in RenCa cells (S2 Fig).

### Dnajb8 has a role in tumor initiation *in vivo*

**Fig 5:**
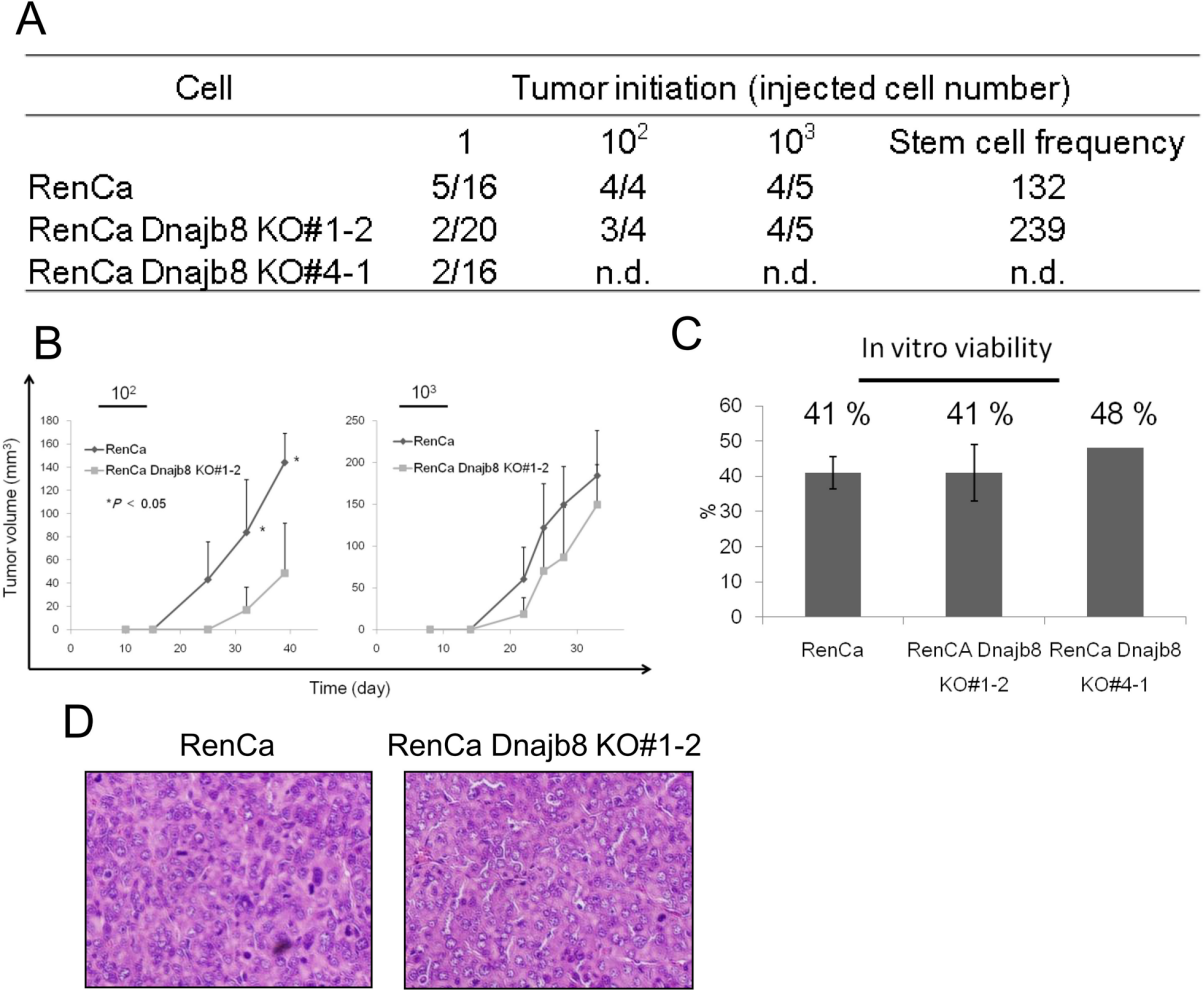
*In vivo* analysis of stemness. (A) Summary of tumor initiation. Tumor-initiating abilities were evaluated at day 33 after injection of 10^3^ cells, at day 39 after injection of 10^2^ cells and at day 70 after injection of single cells. (B) Tumor growth curves of WT and Dnajb8 KO cells. Cells were inoculated subcutaneously into the backs of five Balb/c mice and tumor growth was measured weekly. Data represent means ± SD. The difference between wild RenCa cells and two KO RenCa cells was examined for statistical significance using Student’s *t*-test. (C) Compensated tumor initiation rate for single cell injection. Data was obtained by calculation with alive cell rates *in vitro* culture. (D) Histological photos of tumors derived from WT cells and Dnajb8 KO cells. Magnification, × 200.

To address the tumor initiation ability of Dnajb8 KO cells, syngeneic transplantation was performed. One ×10^3^ and 1 ×10^2^ of WT cells and clone #1–2 cells were injected, and there was no significant difference between WT cells and clone #1–2 cells in 1×10^3^ cell injection; however, the tumor growth of clone #1–2 cells was significantly slower than that of WT cells (Fig 5A and 5B). The histology of tumors derived from WT cells and KO cells did not show any significant difference (Fig 5D). Tumors were initiated by WT in all 4 mice by injection of 1×10^2^ cells, and tumors were initiated by clone #1–2 cells 3 of the 4 mice by injection of 1×10^2^ cells (Fig 5A). The tumor initiation rates of RenCa cells were too high to estimate the frequency of CSCs/CICs, and we therefore performed syngeneic transplantation at the single cell level. WT cells and clone #1–2 and clone #4–1 cells were single cell sorted by a flow cytometer, and the single cells were injected into mice. Tumors were initiated in 5 of 16 WT cell-injected mice, 2 of 20 clone #1–2 cell-injected mice and 2 of 16 clone #4–1 cell-injected mice (Fig 5B). To eliminate the possibility of cell death due to mechanical damage by single-cell sorting, single cell sorted cells were cultured *in vitro* and the survival rates of WT, clone #1–2 and clone #4–1 cells were 41%, 41% and 48%, respectively. We therefore calculated the tumor-initiating rates/survival rates as putative tumor-initiating rates, and putative tumor initiation rates were 76%, 24% and 26%, respectively (Fig 5C). Therefore, the ratios of CSCs/CICs were approximately 3 in 4 WT cells, 1 in 4 clone #1–2 cells and 1 in 4 clone #4–1 cells.

## Discussion

Gene targeting by homologous recombination and that by RNA interference (RNAi) are two major approaches to analyze gene functions [21, 22]. Complete gene KO can be achieved by gene targeting; however, gene targeting by homologous recombination needs much effort, time and cost. Recent genome engineering technologies including ZEN, TALEN and CRISPR/Cas9 enable easy time-saving high-throughput gene targeting [15]. RNAi using siRNAs is also available for high-throughput gene knockdown; however, gene knockdown is temporary and incomplete. In a previous study, we analyzed the function of DNAJB8 by gene knockdown using siRNAs and gene overexpression, and found that DNAJB8 has a role in the maintenance of RCC CSCs/CICs [10]. In that study, we observed that DNAJB8 knockdown suppressed tumor growth; however, tumor growth was also observed in mice injected with DNAJB8 knockdown ACHN human RCC cells. We could not conclude whether the tumor initiation observed in DNAJB8 knockdown cells were due to incomplete gene knockdown or whether DNAJB8 has a partial role in the tumor initiation. In this study, we established DNAJB8 knockout clone lines #1–2 and #4–1 by using the CRIPR/Cas9 system. The efficiency of KO cell establishment was not as high as that in a previous study using embryonic stem (ES) cells [23]. The difference might depend on the type of cells, and further accumulation of experience might reveal the factors that regulate the efficacy of gene targeting using the CRISPR/Cas9 system. In this study, DNAJB8 KO cells showed no DNAJB8 protein expression, and those cells showed reduced tumor initiation ability but could still initiate a tumor as revealed a single cell injection assay. Thus, these results indicate that DNJB8 has a role in the maintenance of CSCs/CICs; however, DNAJB8 is not a single key factor and there must be other key factors.

Several genes have been reported to be related to tumor initiation of CSCs/CICs, and most of them are transcription factors or transcription repressors. The SOX family consists of transcription factors containing an HMG of DNA-binding domains that is expressed in a wide variety of tissues and has important roles in the regulation of organ development and cell-type specification. SOX2 is expressed in ES cells, neural stem cells and normally differentiated gastric epithelial cells, suggesting that it has various functions. In neural stem cells, SOX2 acts with SOX1 and SOX3 to maintain the self-renewal ability of cells [24]. Knockdown of SOX2 using siRNAs abrogated the tumor initiation of lung adenocarcinoma CSCs/CICs [18]. Notch signaling promotes the maintenance and proliferation of non-neoplastic neural stem cells and obstructs differentiation [25]. It was reported that the Notch pathway is activated in CSCs/CICs of many cancer cell lines. Inhibition of the activity of γ-secretase decreased tumor initiation, and overexpression of NICD2, which is one of the Notch signaling factors, enhanced tumor initiation and increased the ratio of CSCs/CICs in medulloblastoma [25, 26]. Bmi-1 belongs to the polycomb gene family and was reported to play a role in the regulation of hematopoietic and neural stem cell self-renewal [27–29]. Moreover, it was reported that knockdown of Bmi-1 using siRNAs inhibited the tumor initiation of oral squamous cell carcinoma CSCs/CICs [30]. Thus, stem cell-related transcription factors have a role in the maintenance of CSCs/CICs. However, the expressions levels of Bmi-1 and Klf4 were same in RenCa WT cells and Dnajb8 KO cells. And, Sox2 and Nanog were not detected. Thus, these transcription factors might not related to the maintenance of CSCs/CICs by Dnajb8. It is still not clear whether DNAJB8 has transcriptional activity; however, a previous study revealed that DNAJB8 interacts with histone deacetylases (HDACs) [7]. DNAJB8 localizes in the cytoplasm of HeLa cervical cancer cells [8]; however, we observed that some of DNAJB8 can localize in the nucleus of HEK293 cells (data not shown). Thus, nucleus-localizing DNAJB8 may regulate the gene expression of stem cell-related transcription factors by associating with HDACs. The incomplete inhibition of tumor initiation by syngeneic transplantation suggests that regulation of gene expression by DNAJB8 may not be as solid as that by stem cell-related transcription factors.

DNAJB8 belongs to the HSP 40 family and has a role in protein folding [7, 8]. In this study we performed similar assay using polyQ construct, but no protein aggregation was observed in RenCa. The different results might depend on the type of cells used in assays. It was reported that HSPs and heat shock factor-1 (HSF1) have essential roles in tumor initiation of cancer [31]. CSCs/CICs were reported to be resistant to several treatments [3]. We thus hypothesized that DNAJB8 has a role in stress resistance of CSCs/CICs. However, our results indicate that DNAJB8 is dispensable for stress responses except for docetaxel, and another factor may be responsible for stress resistance including resistance to chemotherapy and radiotherapy.

In summary, we established and analyzed two Dnajb8 KO RenCa cell lines by using the CRISPR/Cas9 system. Dnajb8 has a role in sphere formation, ratio of SP cells and tumor initiation; however, it is dispensable for stress responses. These results provide important information on the maintenance of CSCs/CICs.

## Supporting Information

S1 Fig RT-PCR of stem cell-related genes. The expressions of stem cell related genes (*Sox2*, *Nanog*, *Klf4*, *Bmi1*) were addressed by RT-PCR. *Gapdh* was used as an internal positive control.
(TIF)

S2 Fig Transfection of Ataxin-3 T-Q82 gene. RenCa, Dnajb8 overexpressed RenCa (RenCa/Dnajb8), Dnajb8 KO cells (RenCa/Dnajb8 KO #1–2 and #4–1) cells were transfected with Ataxin-3 T-Q82 gene fused with GFP protein. The overexpressed Ataxin-3 T-Q82/GFP protein were visualized by confocal laser microscopy. DAPI was used for nuclear staining.
(TIF)
