# Supplementary figures and images for: Dnajb8, a Member of the Heat Shock Protein 40 Family Has a Role in the Tumor Initiation and Resistance to Docetaxel but Is Dispensable for Stress Response
Source: PLoS One. 2016 Jan 11;11(1):e0146501. doi: 10.1371/journal.pone.0146501 (PMC4709114; doi:10.1371/journal.pone.0146501)

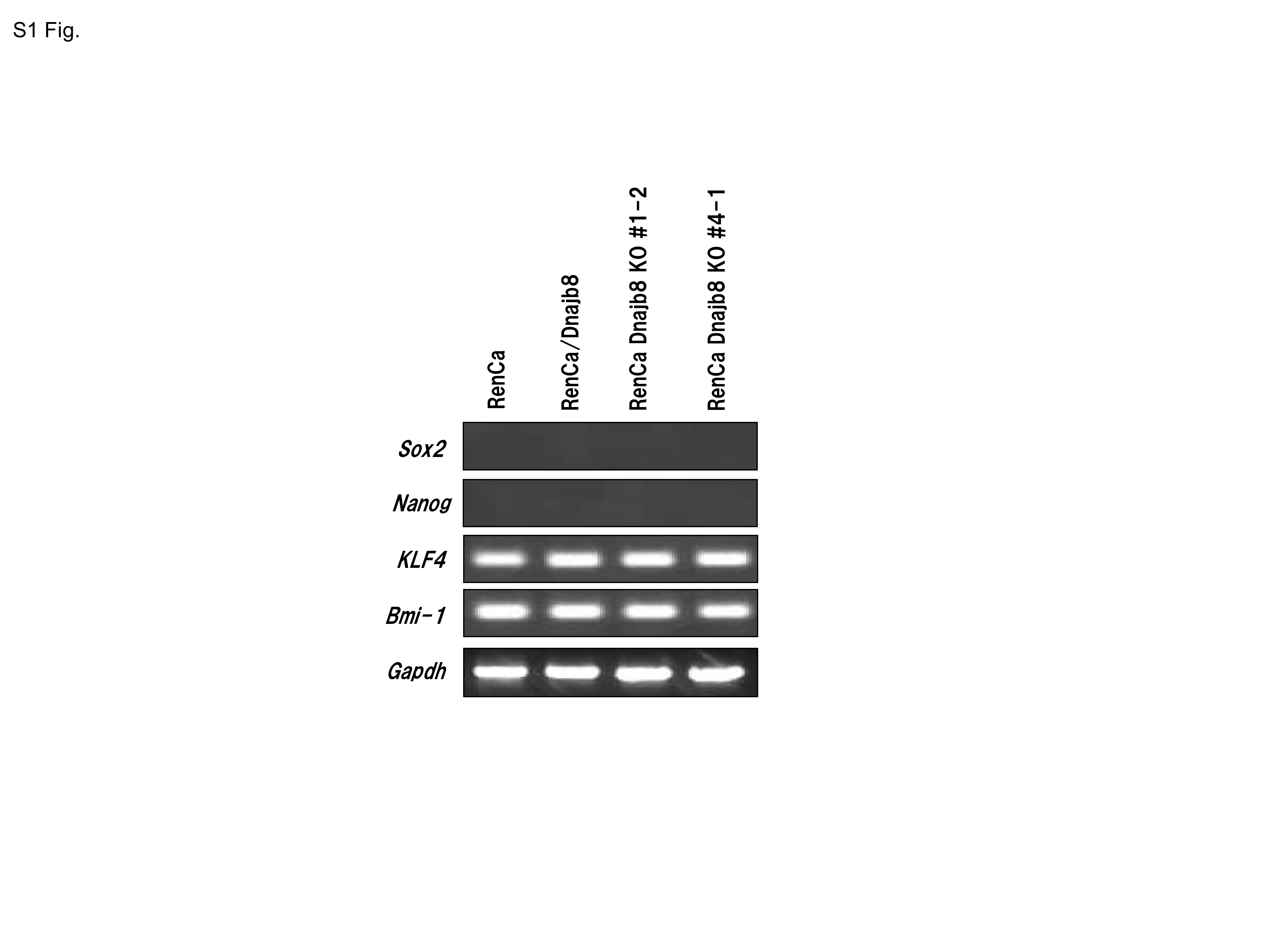

Supplement: S1 Fig — The expressions of stem cell related genes (Sox2, Nanog, Klf4, Bmi1) were addressed by RT-PCR. Gapdh was used as an internal positive control. (TIF) [file pone.0146501.s001.tif]

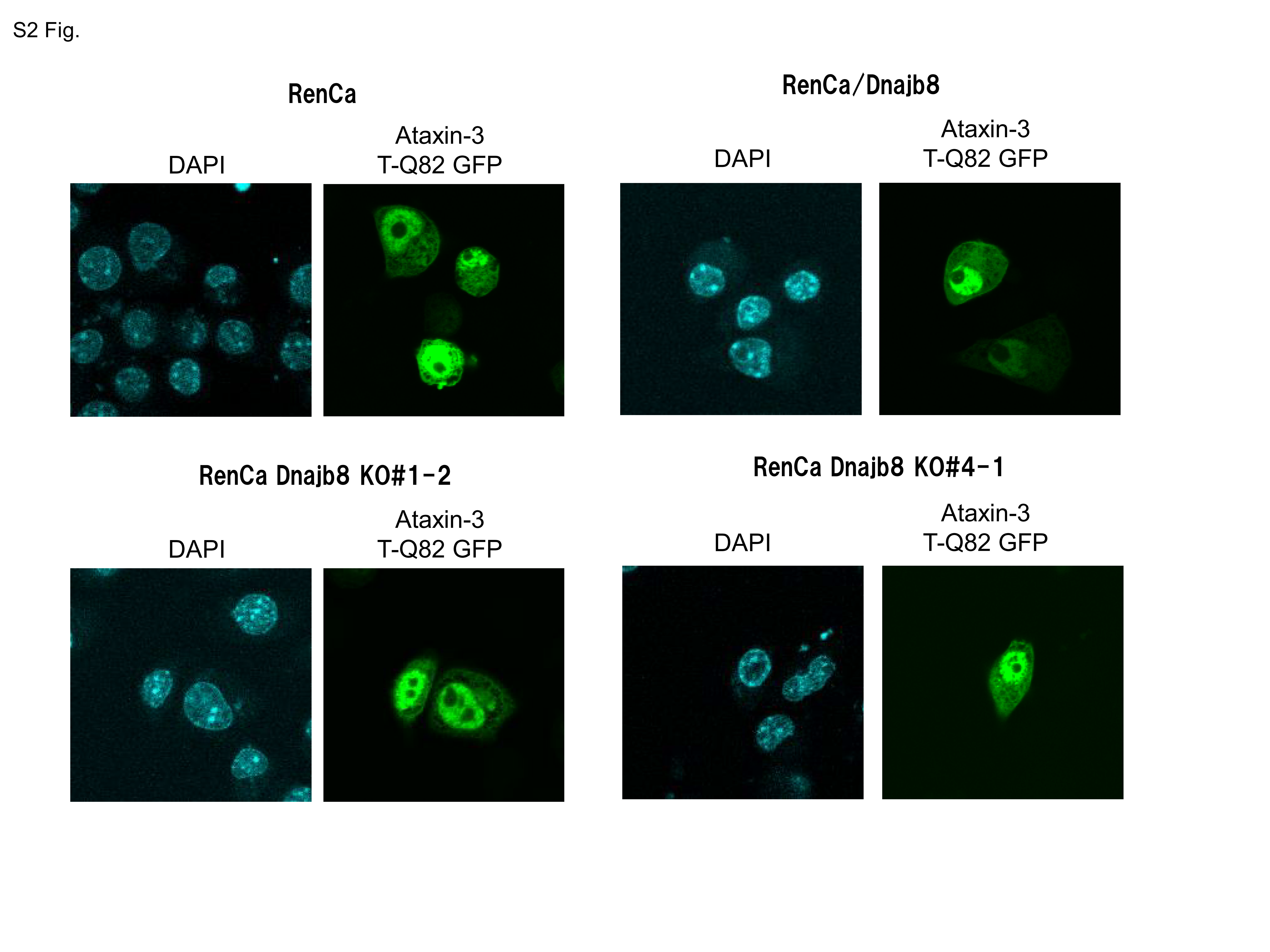

Supplement: S2 Fig — RenCa, Dnajb8 overexpressed RenCa (RenCa/Dnajb8), Dnajb8 KO cells (RenCa/Dnajb8 KO #1–2 and #4–1) cells were transfected with Ataxin-3 T-Q82 gene fused with GFP protein. The overexpressed Ataxin-3 T-Q82/GFP protein were visualized by confocal laser microscopy. DAPI was used for nuclear staining. (TIF) [file pone.0146501.s002.tif]
